# Salat dhuha effect on oxidative stress in elderly women: A randomized controlled trial

**DOI:** 10.1016/j.sjbs.2023.103603

**Published:** 2023-02-23

**Authors:** Elman Boy, Aznan Lelo, Sagiran

**Affiliations:** a Department of Public Health, Faculty of Medicine, Universitas Muhammadiyah Sumatera Utara, Medan, Indonesia; b Department of Medicine and Therapeutics, Faculty of Medicine, Universitas Sumatera Utara, Medan, Indonesia; c Department of Surgery, Faculty of Medicine, Universitas Muhammadiyah Yogyakarta, Yogya, Indonesia

**Keywords:** Salat Dhuha, Glutathione Peroxidase, Malondialdehyde, Elderly, Oxidative Stress

## Abstract

**Background:**

The aging process and a chronic sedentary lifestyle in the elderly as a result of physical restrictions during the COVID-19 pandemic, induces oxidative stress through oxygen supply and antioxidant activity imbalance which in turn induce degenerative diseases. Salat dhuha as a prayer and mind–body medicine which is practiced by the Muslim community can hopefully be a solution to decrease oxidative stress in the elderly.

**Objective:**

To evaluate the acute physiological effects of salat dhuha on Glutathione Peroxidase activity (GPx) as an antioxidant and Malondialdehyde (MDA) as an oxidant in healthy elderly Muslim women population who have done salat dhuha regularly.

**Method:**

A randomized controlled study was done on elderly women (aged 60–74 years old) who are treated in the North Sumatra Government's Nursing Home in Binjai and who routinely do 2 rakaat of salat dhuha every day. Several physical, clinical, and blood examinations were done pre and post-intervention. 101 elderly Muslim women in the nursing home were selected, 26 met the study criteria and were included in the study. The volunteers were randomized into 2 groups using lottery papers, the “2-rakaat salat dhuha group” (n = 13) and the “8-rakaat salat dhuha group” (n = 13). All volunteers did salat dhuha for at least 5 days per week for 6 weeks.

**Result:**

24 elderly women completed the study, and one volunteer from each group dropped out. The characteristics of participants from both groups were homogenous. Results of the *t*-independent analysis showed that MDA concentrations in both groups in the pre and post-test were not significantly different (p > 0,05). Mann Whitney analysis showed that GPx on both groups in the pre and post-test were not significantly different (p > 0,05). Paired sample *t*-test analysis on the MDA concentrations pre and post-test in the 8-rakaat group showed a significant difference in MDA levels (p < 0,05). The 8-rakaat salat dhuha group showed that GPx activity increases as much as 8,9% and MDA levels decrease as much as 48,35 % after 6 weeks.

**Conclusion:**

Salat dhuha promotes redox homeostasis and has the potential to prevent oxidative stress in elderly women.

## 1 Introduction

During physiological conditions, oxidants and antioxidants are in a state of balance, but an increase of reactive oxidative stress (ROS) at the cellular level in the human body as a result of a sedentary lifestyle and stress exposure will affect chronic diseases on the elderly (Bakadia et al., 2021). ROS is provoked by an imbalance between oxidants and antioxidants in the mitochondria (Lauridsen, 2019). ROS is an imbalance between prooxidant formation and the ability of the body's oxidant to decrease or eradicate the dangerous effects of prooxidants (Varesi et al., 2022). There are many types of oxidants, including malondialdehyde (MDA). MDA is one of the most popular oxidant biomarkers in clinical and biomedical settings (Mas-Bargues et al., 2021). MDA is one of the most common and most dangerous lipid peroxides which can cause cell damage and reacts with free amino acid groups through DNA mutagenic activity on its target location, guanine (Barrera et al., 2018). Therefore, MDA can be used to estimate the oxidative stress intensity or damage caused by lipid peroxide. GPx is an important cellular antioxidant since its role is to protect cells from oxidants due to detoxifying lipid peroxidase and hydroperoxide through a catalytic process and recycles vitamins C and E (Ali et al., 2020, Huang et al., 2018).

Oxidative stress is a key pathogenic process that causes tissue injury or cell death, especially through apoptosis or necrosis involved in the pathogenesis of a variety of biological systems (Kruk et al., 2019). ROS not only role as signaling molecules in physiological processes but also play in pathological processes (Alfadda and Sallam, 2012). Increased ROS due to chronic sedentary behavior for the elderly will provoke oxidative damage to mitochondrial tissue, especially complex I, II, and ATP so that the ability of the mitochondrial matrix to produce energy and antioxidants is disturbed (Mikhed et al., 2015). Constantly mild and moderate intensity physical activity may induce increasing antioxidants through activation of transcription factors for the expression of antioxidant nuclear factor erythroid 2 related factor (NRF2) due to ROS stimulation. This transcription factor may induce the expression of GPx for protecting human muscle fibers from damage due to ROS (He et al., 2016).

The benefits of consistent physical activity on physical and mental health are unquestionable, but for elderly people, it still has to be carefully implemented it. Improper physical activity can provoke the risk of disease and injury for the elderly (Langhammer et al., 2018). During Covid-19 pandemic, physical activity was very limited, and choosing the right physical activity for the elderly was very important (Callow et al., 2020). A young people, moderate-intensity physical activity will push the oxidative stress accident, but in the elderly population, the results are still inconsistent (Tan and Siah, 2022). Whether physical activity may stimulate antioxidant enzyme activity in young but elderly people the result is still a mystery (Muhammad and Allam, 2018).

Practitioners and researchers have seen that physical activity with a spiritual approach called mind–body medicine may be used as a solution to improve human body fitness (Saniotis, 2018). Salat is a mind–body medicine that comes from Islam. Salat contributes to improving elderly women's blood pressure. Salat comes from the Arabic word which means prayer and it is an important practice of worship in Islam (Hidayat, 2017). Salat is a combination of mild to moderate physical activity. Salat is a systematic movement of the body while praying to God (Allah) which is called 'rakaat' (Osama and Malik, 2019). Salat dhuha is a recommended praying to do for Muslims. Salat dhuha is performed in the morning, which starts 15 min after sunrise and until midday. The number of rakaat of salat dhuha are 2, 4, 6, 8, and 12 rakaat (Munthe et al., 2021).

Previous studies stated that salat has numerous benefits for physical and spiritual health (Chamsi-Pasha and Chamsi-Pasha, 2021). For elderly women, salat dhuha 8 rakaat for 6 weeks has proven more significant effect on improving blood pressure and heart rate compared to only 2 rakaat in the same timeframe (Boy et al., 2021). For more than a decade, various studies have reported the effect of salat on human body health, but no research has shown the effect of salat dhuha against ROS for elderly women. Therefore, this study's objective is to analyze the effect of salat dhuha on GPx and MDA activity in healthy elderly women.

## 2 Materials and Method

### 2.1 Study population

**Fig. 1:**
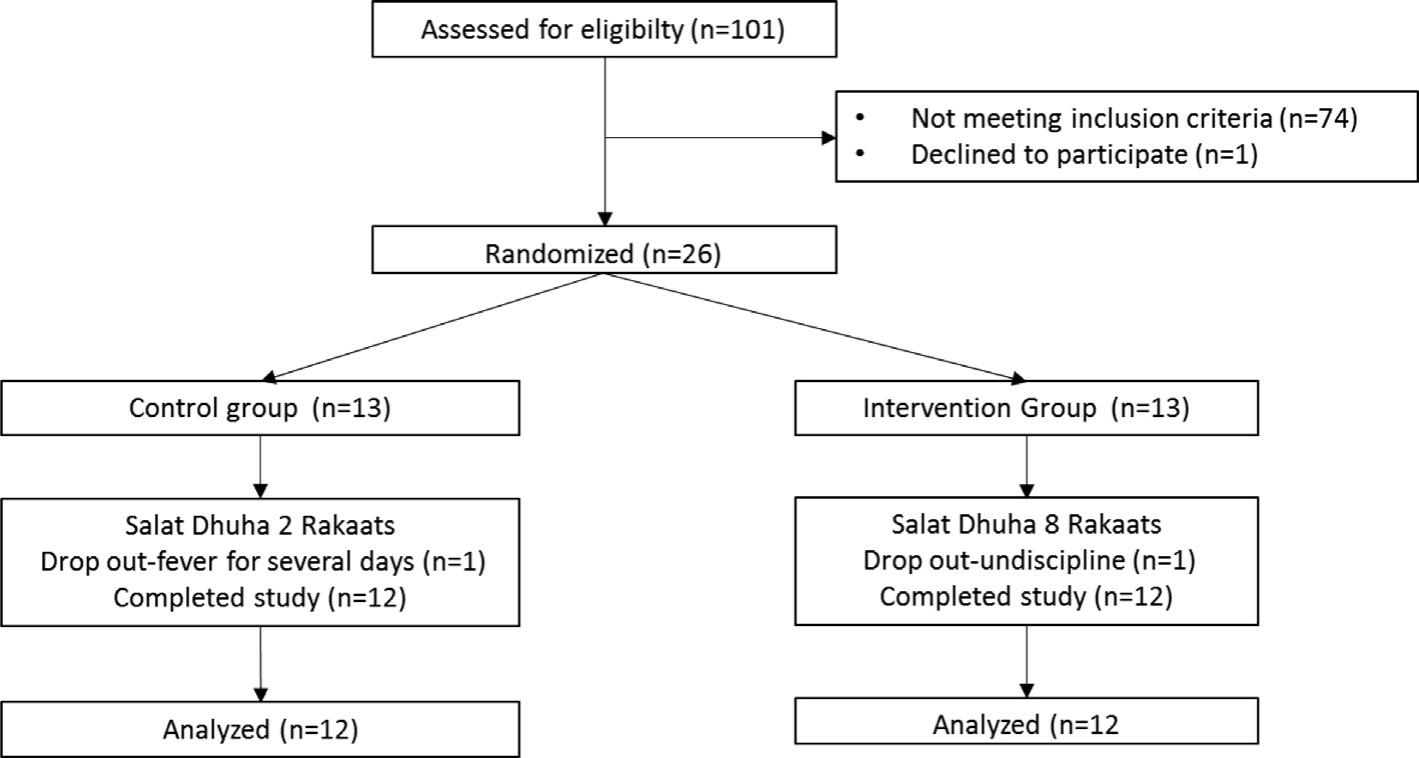
Consort flow diagram.

101 elderly women in North Sumatra Nursing Home in Binjai followed the selection, and 26 volunteers met the study criteria. The criteria are: aged 60–74 years old, routinely performs salat dhuha with at least 2 rakaat each day, has good physical health, not a diabetic, not hypertension, not a smoker, not consume medication, and not overweight (body mass index < 30 kg/m2). To ensure that each subject was in a good health, they have to pass a medical check-up before recruitment. The sample size has been calculated and meets the minimum size for paired numerical comparative equations, and repeated measurements with two measurements (Dahlan, 2020). Volunteers were divided into two groups randomly using lottery papers. The volunteer who had taken number 2 was included in “2 rakaat of salat dhuha group” (13 volunteers) and who had taken number 8 were included in “8 rakaat of salat dhuha group” (13 volunteers) (Fig. 1). During the study, all subjects were asked not to change their daily habits including their physical activity. If they change their daily habits, they have been asked to report it to the researcher dropouts were not included in the analysis. This study has received consent from Universitas Sumatra Utara (USU) ethics committee (Ethic Code 776/KEP/USU/2020). All volunteers have given their consent before being recruited in this study. (See Fig. 1)

### 2.2 Salat dhuha

Every subject should participate in the salat dhuha congregation in the mosque and be led by an imam who has certification as an imam from the Indonesian Ulema Council. Salat dhuha was held for 6 weeks, 5 times a week, between 08.00 and 10.00 Western Indonesian Time (WIB). 8 rakaat group of salat dhuha congregation prayed in the first row, while the 2 rakaat group was behind them. The salat dhuha be practiced in some cycles. Each cycle consists of two rakaat (2 rounds) salat dhuha. Each rakaat/round consists of 7–9 different postures (Koubaa et al., 2020). During salat, all worshipers were required to recite verses from the Quran (moving their lips but keeping silent). First rakaat begins with qiyam (a standing position and the initial takbir, raising both hands to ear level, then lowering them and the right arm holding the left arm above the chest for 60–90 s). Then, rukuk or bowing (moving the spine forward, especially in the lumbar joints, is supported by two straight hands gripping the outstretched knees. This is done for 5–10 s). Then iktidal (stand upright for 3–5 s). then, sujud or prostrate (kneel and place your forehead on the floor, with your palms at ear level, for 5–10 s). Then sit on both feet, then prostrate again. Thus the first round of salat dhuha has been completed. Then the congregation stands again to start the second round. Start again with qiyam, bowing, iktidal, prostrate, sitting on the feed, then prostrate again. Until here the time used for each movement is the same as the first round. Then proceed to sit with the left knee bent with the ankle bent in reverse dorsi and the right knee and right metatarsophalangeal joint bent for 60–75 s, then ending with a greeting, looking at the right shoulder once and one left shoulder times while saying a salat of salvation (Jamari et al., 2017).

### 2.3 Study design

This study is a nonblinded randomized controlled study. This study is aimed to analyze the effects of salat dhuha on GPX activity as an antioxidant and MDA as an oxidant in healthy elderly women. For the biomarker analysis, peripheral blood serum taken from the volunteer's right arm was collected pre and post (week-0 and week-6) using a BD Vacutainer blood-collecting tube and was stored at −80 °C. Volunteers in the “2-rakaat salat dhuha group” kept performed 2 rakaat of salat dhuha, while the “8-rakaat salat dhuha group” performed 8 rakaat of salat dhuha (Table 1).

**Table 1:** Salat Dhuha Rakaat and Activities.

Program	Activities
2-rakaat salat dhuha	*Takbir (stands) → qiyyam (stands) → ruku (bowing) → qiyyam (stands) → sujud (prostrates) → tashahud (sits) → sujud (prostrates) → qiyyam (stands) → ruku (bowing) → qiyyam (stands) → sujud (prostrates) → tashahud (sits) → sujud (prostrates) → tashahud akhir (sits) → salam (turns head)*
8-rakaat salat dhuha	*Takbir (stands) → qiyyam (stands) → ruku (prostrates) → qiyyam (stands) → sujud (prostrates) → tashahud (sits) → sujud (sits) → qiyyam (stands) → ruku (prostrates) → qiyyam (stands) → sujud (prostrates) → tashahud (sits) → sujud (prostrates) → tashahud akhir (sits) → salam (turns head).* This cycle have been done 4 times.

### 2.4 Confirmation on serum Malondialdehyde concentration

MDA is examined with a Thiobarbituric Acid Reactive Substances (TBARS) assay kit and spectrophotometry with a 535 nm wavelength (Moselhy et al., 2013). The average lipid peroxide serum is determined by a Human Quantitative Determination of Thiobarbituric Acid Reactive Substances, QuantiChromTM, and TBARS Assay Kit (DTBA-100). The supernatant sample obtained from 100 µl serum and added with cold 200 µl 10 % trichloroacetic acid (TCA) is incubated for 5 minutes above the ice, then centrifuged with a 14.000 rpm speed for 5 min (dilution factor n = 3). The standard solution was obtained from 4 µl 6 M MDA standard added with 2396 µl ddH20 (end concentration 10 mM MDA). 10 mM MDA is diluted with 997 µl ddH20 (gets a 30 µl MDA concentration). Then, 30 µl MDA is diluted with H20 to produce 30 µl, 18 µl, 9 µl, and 0 µl dilution standards to count the average MDA serum.

### 2.5 Confirmation on serum Glutathione peroxidase activity levels

The activity levels of glutathione peroxidase are assessed with Glutathione Peroxidase Assay Kit, Calbiochem®, catalog number: 353919. GPx activity is measured indirectly with a combined reaction with glutathione reductase (GR). In short, all wells background, or non-enzymatic, wells positive control (bovine erythrocyte glutathione peroxidase) and wells samples are added with 20 µl Cumene Hydroperoxide. Then the plate is shaken carefully for a few seconds. Absorbance is read each minute on 340 nm wavelength 5 times with spectrophotometry, optical density count (OD), then GPx activity count with a formula that has been determined by the kit in nmol/min/ml.

## 3 Data analysis

The statistical core comparison between both groups and the change in score between both groups were analyzed with a *t*-test. Significant statistic levels were reported in p < 0,05. Changes in result measurements (Δ) between both groups were tabulated in percentage (%) which was calculated with:

$$=\frac{outcome\;post\;6\;weeks\;intervention\;-\;outcome\;0\;week\;\;intervention}{outcome\;0\;week\;intervention}\;\times \;100\%$$

## 4 Result

### 4.1 Basic characteristics

Twenty-four volunteers (12 in the '2-rakaat salat dhuha group“ and 12 in the 8-rakaat salat dhuha group”) have completed the study. One volunteer from the 2-rakaat salat dhuha group and one volunteer from the 8-rakaat salat dhuha group dropped out from the study from being sick and were treated in a hospital as a result of a disease that wasn't related to this study. The basic characteristics of the volunteers who have done this study were homogenous (no difference between both groups), whether on the number of rakaat, age, cholesterol levels, blood sugar levels, hemoglobin levels, systolic blood pressure, diastolic blood pressure and heart rate (Table 2).

**Table 2:** Mean ± SD basic characteristics based on groups.

Feature	Salat Dhuha Group		*P*
	2 Rakaat	8 Rakaat	
	Mean ± SD	Mean ± SD	
Salat dhuha	2,17 ± 5,77	2	0.31b
Age (years)	68,08 ± 3,80	68,58 ± 4,62	0.77b
Height (cm)	147,92 ± 4,94	150,50 ± 4,05	0.17a
Weight (kg)	48,64 ± 5,55	46,72 ± 4,41	0.35b
BMI (kg/m2)	22,23 ± 2,63	20,65 ± 2,08	0.11a
Cholesterol (mg/dL)	158 ± 32,90	172 ± 25,77	0.25a
Blood Glucose1-2 h PP (mg/dL)	112 ± 19,40	118 ± 20,37	0.50a
Hemoglobin (g/dL)	13,12 ± 1,52	13.20 ± 1,27	0.88a
Systolic blood pressure (mmHg)	128,75 ± 4,11	127,25 ± 7,49	0.81b
Diastolic blood pressure (mmHg)	78,08 ± 7,42	77,83 ± 7,15	0.93a
Heart rate (x/mnt)	80,08 ± 4,56	83,91 ± 10,50	0.25a

a = Independent *t*-test (P Shapiro Wilk value > 0,05); b = Mann whitney (P Shapiro Wilk value < 0,05); P Value > 0,05 → no significant difference.

### 4.2 Effects on MDA levels and GPx characteristics

Table 2 shows the characteristics of the two groups were homogeneous (P > 0.05). The independent *t*-test analysis of MDA levels in the two groups did not show a significant difference (P > 0.05). Mann Whitney analysis on GPx activity also shows P > 0.05.

Table 3 shows the results of pre-test MDA levels in both groups were homogeneous (P > 0.05). The results of the post-test MDA levels in both groups were also homogeneous (P > 0.05). MDA levels in the 2 rakaat of the salat dhuha group increased, although not significantly (P > 0.05), but in the 8 rakaat of the salat dhuha group, MDA levels decreased significantly (P < 0.05). The results of the pre-test and post-test GPx activity examination in both groups were homogeneous (P > 0.05). After 6 weeks of intervention, the results of examining GPx activity in the 2 rakaat of the salat dhuha group decreased. However, in 8 rakaat of the salat dhuha group, GPx activity indicated a significant increase (P < 0.05).

**Table 3:** Mean ± SD and P value of MDA levels and GPX activities on both groups.

Feature	Salat Dhuha Group		*P*
	2 Rakaat	8 Rakaat	
	Mean ± SD	Mean ± SD	
Malondialdehyde (µM)			
Pre	0,55 ± 0,16	0,91 ± 0,57	0.06a
Post	0,69 ± 0,42	0,47 ± 0,28	0.14a
*P*	0.31b	0.04b*	
Glutathione peroxidase (nmol/min/ml)			
Pre	232,53 ± 39,95	193,49 ± 60,36	0.18b
Post	217,24 ± 34,15	210,74 ± 40,61	0.67a
*P* value to compare each group’s pre and post test	0.08 d	0.32c	

a = independent *t*-test; b = mann whitney; c = dependent *t*-test; d = Wilcoxon; *P Value < 0,05 → significant difference.

**Fig. 2:**
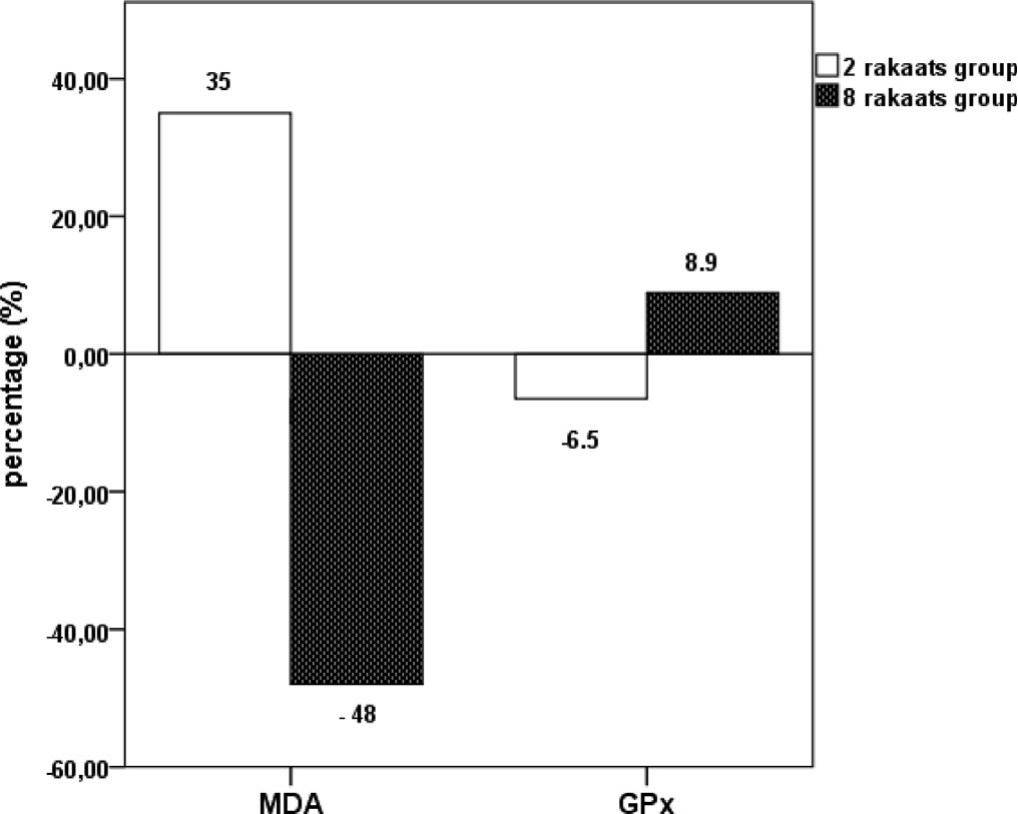
The percentage difference of malondialdehyde (MDA) and Glutathione peroxidase (GPx) in the 2 rakaat and 8 rakaat salat dhuha groups after 6 weeks.

Fig. 2 shows that the 8 rakaat of the salat dhuha group succeed to reduce MDA levels by as big as 48 % and increase GPx activity by as big as 8.9 %. However, in the 2 rakaat of the salat dhuha group, the contrary situation happened. Here MDA levels increased by 35 % while GPx activity decreased by 6.5 %.

## 5 Discussion

Subjects recruited in this study were healthy volunteers with homogenous characteristics. Subject randomization can only be done in a homogeneous population. Clinical trials with heterogeneous interventions or populations often produce confusing results (Husain and Srijithesh, 2016). This is the first study that examines the effects of salat dhuha for 6 weeks on oxidative stress in elderly Muslim women. The results of this study confirmed the position of salat dhuha as one of the most important mind–body medicine and should be held consistently by every Muslim, especially elderly women. Even though previous studies on the effects of salat against oxidative stress don't exist, a lot of studies related to mind–body medicine against oxidative stress have been done. For example, studies on yoga which were correlated to oxidative stress in elderly men (aged 60–80) with grade I hypertension, through a randomized controlled study in China found that yoga intervention done for 1 hour in the morning, 6 hours a week for 3 months decreased serum MDA levels significantly as much as 20 % (p < 0,01) (Yu et al., 2018). Data on the characteristics of the subjects in the pre-intervention showed that the volunteers in the 2 rakaat of salat dhuha group had routinely performed>2 rakaat of salat dhuha before this study began. Then these habits must be customized since they were randomly selected to participate in the 2 rakaat of the salat dhuha group. This study shows that degrade of routine rakaat number of salat dhuha will provocate ROS in the human body. This happens because sedentary will increase systemic inflammation and oxidative damage at the cellular level (Sofra and Badami, 2020).

Free radicals (ROS) not only act as signaling molecules in physiological processes but also play a role in pathological processes (Alfadda and Sallam, 2012). Increased oxidative stress due to chronic sedentary behavior in the elderly will cause oxidative damage to mitochondrial tissue, especially complex I, complex II, and ATP complex so that the ability to form energy and the ability of the mitochondrial matrix to produce antioxidants to counteract free radicals is disrupted (Mikhed et al., 2015). Continuous mild and moderate intensity physical activity will lead to adaptation by increasing antioxidants through activation of transcription factors for the expression of antioxidant nuclear factor erythroid 2 related factor (NRF2) due to ROS stimulation. This transcription factor will increase the expression of GPx which plays a role in protecting muscle fibers from damage due to ROS (He et al., 2016). Antioxidants will neutralize the overproduction of oxidants including turning oxidants into something harmless. One of the antioxidants that play a vital role in the human body is GPx (Ali et al., 2020).

Salat dhuha with more rakaat is correlated to MDA level reduction and an increase in GPx activity. The result of this study showed that 8 rakaat of salat dhuha conducted for 6 weeks was more effective in increasing GPx activity and in decreasing MDA concentration compared to 2 rakaat of salat dhuha in elderly Muslim women. This study shows that increasing the number of salat dhuha rakaat could grow up GPx activity and push down MDA levels in human blood serum. The specific reason for this consistency can be explained through the antioxidant's dynamic response toward ROS. Several studies showed that oxidation levels are more stable or decrease from Tai chi exercise Each rakaat of salat dhuha takes around 2 minutes, as a result, 2 rakaat of salat dhuha takes 20 minutes per week, while 8 rakaat of salat dhuha takes around 80 minutes per week, this emphasizes enough time for physical activity done regularly. The effects of physical activity and aerobic capacity volume against oxidative stress have been confirmed by previous studies (Kruk et al., 2019). A decrease in MDA concentration is correlated to an increase in GPx activity which indicates that GPx has reduced ROS, which is produced by physical activity and age (da Silva et al., 2019).

Previous studies stated that GPx activity will only increase with moderate physical activity intensity if done for>8 weeks. This research has provided new information that salat dhuha which is performed for 6 weeks can increase GPx activity. Undoubtedly, 8 rakaat of salat dhuha as a physical and spiritual activity gives an acute adaptive effect which is an increase in antioxidant enzyme levels. Low and moderate-intensity physical activity which is done consistently is effective in increasing GPx activities through transcription factor activation, antioxidant expression, and nuclear factor erythroid 2-related factors (NRF2) (Vargas-Mendoza et al., 2019). GPx is a very important cellular antioxidant because of its role in detoxifying lipid peroxidase and hydroperoxide through a catalytic process and recycles vitamins C and E to protect cells from oxidants Glutathione prevents damage to important cellular elements, such as control and structural proteins, membrane lipids, and DNA that is provoked by ROS (Al-Sowayan, 2020). Gluthathione prevents damage to important cellular elements, such as control and structural proteins, membrane lipids and DNA that is provoked by ROS (Kruk et al., 2019). Antioxidants also prevent ROS from inducing dysfunction and degradation of skeletal muscle protein pathways, protecting them from muscle atrophy in humans with chronic disease (Moulin and Ferreiro, 2017).

This data shows that physical activity from salat dhuha that is performed regularly is an important factor in maintaining the health status of elderly women. The results of this study are consistent with the redox homeostasis theory that is body's cellular efforts protect cells from oxidation during exercise. Repeated mild and moderate-intensity physical activity produces the body's adaptive response to repair oxidative damage. Moderate-intensity physical activity produces more antioxidants and plays an important role in the mechanism of protection against oxidative stress due to intermittent rest periods (Cheung et al., 2018). Physical activity may also increase ROS thru intrinsic or extrinsic pathways. Intrinsic factor like a change in cellular biochemistry structure which stimulates the production of oxidants, cellular proliferation, and protein synthesis during physical activity, causing muscle fragility and an increased risk of mechanical injury, extrinsic factor is correlated to an overload of physical activity (Larasati and Boy, 2020, Rebelo-Marques et al., 2018, Scicchitano et al., 2018).

This study gives a shred of new evidence that GPx activity increases with mild-moderate intensity physical activity done for 6 weeks. It has been confirmed previously that GPx activity will increase in moderate-intensity physical activity when done for more than 8 weeks. The result of this study showed that mild to moderate intensity physical activity in the form of 8 rakaat of Salat dhuha significantly reduces oxidative stress, leading to better antioxidant protection in elderly women, but future studies need to be done on the effects of salat dhuha which is done for a longer duration, for example, the effects of salat dhuha on oxidant and antioxidant balance in men and women of all ages.

## 6 Conclusion

Salat dhuha promotes redox homeostasis and has the potential to prevent oxidative stress in elderly women.

## Declaration of Competing Interest

The authors declare that they have no known competing financial interests or personal relationships that could have appeared to influence the work reported in this paper.
